# Spatial and temporal distribution dataset of benthic macroalgae during the 2015-2016 tropical monsoonal cycle in Malaysia

**DOI:** 10.3897/BDJ.10.e85676

**Published:** 2022-07-26

**Authors:** Nur Farah Ain Zainee, Mohammad Rozaimi

**Affiliations:** 1 Department of Earth Sciences and Environment, Universiti Kebangsaan Malaysia, Bangi, Malaysia Department of Earth Sciences and Environment, Universiti Kebangsaan Malaysia Bangi Malaysia

**Keywords:** abundance, occurrence, seaweed, specimen

## Abstract

**Background:**

The effects of small-scale disturbances, such as monsoon, are understudied in tropical regions. The storms associated with monsoon events not only modify the local macroalgal community structure, but also reveal the continuation of short-term recolonisation. Thus, this study aims to determine the variation in species, assemblage and cover of macroalgae during the monsoonal cycle from 2015 to 2016. This paper presents data on the spatial and temporal distribution of benthic macroalgae along the coastline of Johor, Malaysia. The information is presented as raw and partially-processed data, which summarises the cover and frequency of macroalgae at the respective study sites. This paper describes an important set of data that can be used further for in-situ experiments on the effects of environmental disturbances towards pioneer and climax species in tropical areas.

**New information:**

This study provides a description of the east coast shore of Peninsular Malaysia, specifically in Johor coast in 2015-2016. The spatial and temporal distribution and abundance of a total of 41 taxa were assessed at four monsoon-exposed locations. These data provide a comprehensive baseline against disturbance and recolonisation of macroalgal community can be effectively and objectively evaluated.

## Introduction

Environmental stress, such as disturbance, occurs over a short period of time and results in significant changes in the ecosystem. Many ecosystems and species evolve in response to particular environmental disturbances that create patches of disturbed habitat and play a significant role in controlling such things as life cycles, food, nutrient supply and habitat availability (Kroeker et al. 2020). Community structure in the disturbed area creates variability at spatial and temporal scales, including in terrestrial, freshwater and marine ecosystems (Turner 2010). In Malaysia, monsoonal storms are the primary annual storms associated with local changes in rainfall, wave, current and wind speed intensities (Satari et al. 2015). Monsoonal storm activities, such as wave impact, heavy winds and strong currents affect the eastern coast of Peninsular Malaysia, specifically Johor which is located in the southern region of Peninsular Malaysia. The coastline features vary from being very exposed to very sheltered and, therefore, impact the delicate marine macrophytes that reside along the coastline, such as macroalgae (Lindenmayer and Fischer 2007).

The existence of macroalgae is usually species-specific as most species require specific conditions for colonising their respective habitats (Zainee et al. 2019b). Dominant macroalgal species can be found attached to any available substrate including in water puddles on the surface of artificial substrate, such as sea wall rocks (Zainee et al. 2019a). However, the effect of monsoon reduces the macroalgal productivity through modification and destruction of their habitat (Kim et al. 2017). These events will not only pull away the early coloniser, fleshy and delicate macroalgal (such as *Chaetomorpa* spp.), but also affect the climax macroalgal community that have strongly-attached holdfasts, such as *Sargassum* (Zainee and Rozaimi 2020). Once the actual disturbance event is finished, the succession process begins and favours those that are opportunistic in nature (e.g. Suding et al. 2004), which may eventually produce a similar ecosystem to the one that existed prior to the monsoon disturbance. Some of the disturbances events relating to macroalgal community are those related to small scale environmental events in general (e.g. Kendrick et al. 2004, Kim et al. 2017, Prathep et al. 2008), whereas others are specific to large scale disturbances such as hurricane and tsunami (Prathep et al. 2008, Wilson et al. 2020). Therefore, more comprehensive studies are needed to obtain data on the impacts of monsoons on the macroalgal community.

Thus, this data paper presents the dataset on the immediate impact of monsoon on the eastern coast of Johor. The temporal and spatial data include the changes in cover and frequency of benthic macroalgae in the area, demonstrating variation in macroalgae diversity over the 14-months study period. Such data allow further in-situ experiments on the effect of environmental disturbances towards pioneer and climax species. Besides, substratum- and habitat-specificity of the macroalgae species is presented, which allows insights into assessing macroalgal abundances. In conclusion, the data serve as part of a larger assessment effort and the dataset synthesises the results of macroalgal diversity work done in the eastern coastal waters of Johor (Malaysia).

## Project description

### Title

Spatial and temporal distribution dataset of benthic macroalgae during the 2015-2016 tropical monsoonal cycle in Malaysia

## Sampling methods

### Study extent

**Figure 1.**
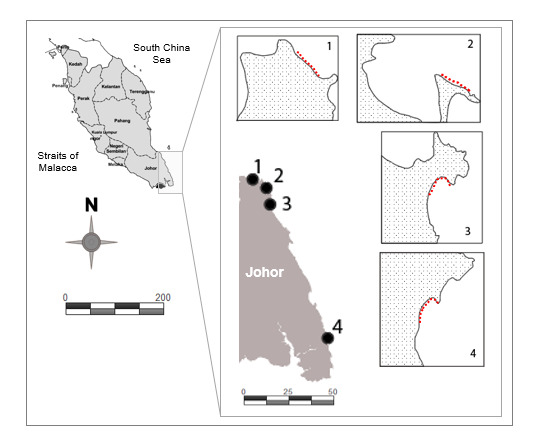
Locations of collected macroalgae along the Johor coast: (1) Pantai Pasir Lanun; (2) Pulau Mawar; (3) Telok Gorek; and (4) Tanjung Lompat.

Sampling activity was conducted in four locations in the eastern Johor coastline: Pantai Pasir Lanun, Pulau Mawar, Telok Gorek and Tanjung Lompat (Fig. 1). Pantai Pasir Lanun is located at the tip of a foreland with a relatively straight coastline, predominantly featuring hard substrates composed of large areas of coral rubble and boulders. Pulau Mawar is characterised by a shallow-elevated sandy terrain with small patches of mangrove trees and coral rubble. Telok Gorek is located within an indented bay, covered with mangrove trees and sheltered from the foreland. Tanjung Lompat consists of a foreland and an extensive bay, characterised by boulder-pebbles on the foreland and a shallow sandy bay.

### Sampling description

**Figure 2.**
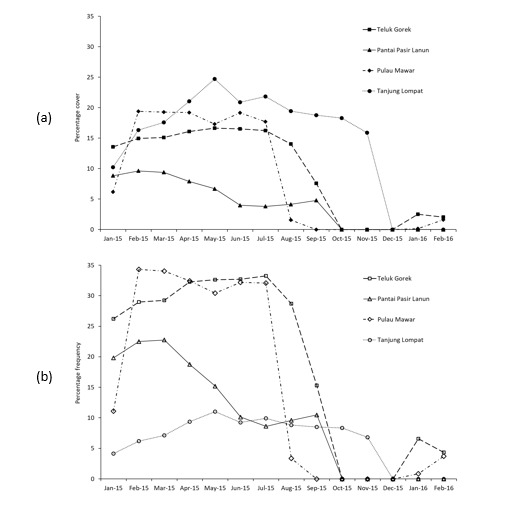
Pre-analytical view of percentage (a) cover and (b) frequency of macroalgae observed at the eastern coast of Johor during 2015-2016 observations.

Sampling was undertaken from January 2015 until February 2016 during the lowest tide of the month (Table 1). Transects were placed randomly, taken to represent the macroalgae cover and frequency at each site. The quadrats were placed alternately at every 1 metre of the 25-metre transect line. Initially, the macroalgae that were found inside the quadrat were recorded, identified and inventoried according to the type of species, percentage of cover and percentage of frequency (Suppl. material 1). The types of substratum attached by macroalgae were noted as representing the habitat specificity of the macroalgae (Table 2). The raw data of cover and frequency were calculated by multiplying the vertical count of every species to the five levels of multiplier and the total number of sub-quadrat from the nine transect lines with a total of 234 quadrats (Suppl. materials 2, 3, 4, 5). The cover of every species of macroalgae was then analysed by summing the percentage cover value of prostrate and erect parts of the macroalgae in each sub-quadrat (10 cm × 10 cm) after Saito and Atobe (1970) (Suppl. material 6). The percentage frequency of macroalgae was obtained by calculating the total number of squares (q_n_) in which the species occurred, divided by the total number of small squares in the quadrat (= 25) and multiplied by 100 (Suppl. materials 2, 3, 4, 5). A pre-analytical view of the percentage cover and frequency data of macroalgae is visualised in Fig. 2.

### Quality control

All scientific names are morphologically identified according to Ismail (1995), Trono and Ganzon-Fortes (1988), Zainee et al. (2018) and Zainee et al. (2019a) and are standardised according to Guiry and Guiry (2021) and WoRMS Editorial Board (2021).

### Step description

1. In-situ identification of species and destructive collection for first-time observed samples and preservation in formaldehyde,
2. Non-destructive sampling (except for filamentous algae that need microscopic observation in the laboratory) at four study sites,
3. Photography, sorting, cleaning and preparation of herbarium specimens,
4. Conversion of paper-based records from the field and laboratory into an electronic data format (Excel spreadsheets),
5. Organising the datasets into a standardised format,
6. Standardisation of taxonomy using the World Register of Marine Species and AlgaeBase,
7. Export of data as a DarwinCore Archive and
8. Generation of dataset-level metadata.

## Geographic coverage

### Description

Sampling was undertaken along four major shore stretches of the entire coast of east Johor, covering approximately 180 km from Desaru to Mersing. The eastern coast of Johor extends approximately 175 km from Teluk Lipat (i.e. Lipat Bay) to the north and Teluk Ramunia to the south.

Coordinates: Pantai Pasir Lanun (02°38'52"N, 103°45'29"E), Pulau Mawar (02°37'08"N, 103°47'01"E), Telok Gorek (02°18'37"N, 103°57'31"E), Tanjung Lompat (01°36'10"N, 104°15'17"E).

### Coordinates

1.197 and 2.757 Latitude; 102.48 and 104.546 Longitude.

## Taxonomic coverage

### Description

We report the identification of marine algae species from rhodophytes, chlorophytes and phaeophytes.

### Taxa included

**Table:** 

Rank	Scientific Name	
class	Ulvophyceae	
class	Phaeophyceae	
class	Florideophyceae	
order	Bryopsidales	
order	Cladophorales	
order	Dasycladales	
order	Ulvales	
order	Dictyotales	
order	Fucales	
order	Ceramiales	
order	Corallinales	
order	Gelidiales	
order	Gigartinales	
order	Gracilariales	
order	Nemaliales	
order	Rhodymeniales	
family	Rhodomelaceae	
family	Lithophyllaceae	
family	Corallinaceae	
family	Pterocladiaceae	
family	Gigartinaceae	
family	Galaxauraceae	
family	Gracilariaceae	
family	Cystocloniaceae	
family	Lomentariaceae	
family	Dictyotaceae	
family	Sargassaceae	
family	Polyphysaceae	
family	Caulerpaceae	
family	Cladophoraceae	
family	Boodleaceae	
family	Ulvaceae	
family	Valoniaceae	
species	*Caulerparacemosa*	
species	*Cladophoropsismembranacea*	
species	*Chaetomorphaaerea*	
species	*Chaetomorphacrassa*	
species	*Chaetomorphaligustica*	
species	*Chaetomorphalinum*	
species	*Chaetomorphaminima*	
species	*Cladophorastimpsonii*	
species	*Cladophoravagabunda*	
species	*Valoniaaegagropila*	
species	*Acetabulariaacetabulum*	
species	*Ulvaclathrata*	
species	*Ulvaintestinalis*	
species	*Dictyopterisdelicatula*	
species	*Canistrocarpuscervicornis*	
species	*Dictyotamertensii*	
species	*Dictyotadichotoma*	
species	*Padina australis*	
species	*Padinaboergesenii*	
species	*Padinaminor*	
species	*Sargassumoligocystum*	
species	*Sargassumpaniculatum*	
species	*Sargassumpolycystum*	
species	*Sargassummicrocystum*	
species	*Sargassumtenerrimum*	
species	*Acanthophoramuscoides*	
species	*Acanthophoraspicifera*	
species	*Polysiphoniacoacta*	
species	*Amphiroafragilissima*	
species	*Janiaadhaerens*	
species	*Pterocladiellacaloglossoides*	
species	*Chondruscrispus*	
species	*Hypneacervicornis*	
species	*Hypneaspinella*	
species	*Gracilariaarcuata*	
species	*Gracilariabursa-pastoris*	
species	*Crassiphycuschangii*	
species	*Gracilariacoronopifolia*	
species	*Gracilariasalicornia*	
species	*Galaxaurarugosa*	
species	*Ceratodictyonintricatum*	

## Temporal coverage

### Notes

2015-01-10 through 2016-02-26

## Collection data

### Collection name

Plantae

### Specimen preservation method

dried and pressed, microscopic preparation

## Usage licence

### Usage licence

Open Data Commons Attribution License

### IP rights notes

To the extent possible under law, the publisher has waived all rights to these data and has dedicated them to the Open Data Commons Attribution License, which permits unrestricted use, distribution and reproduction in any medium, provided the original author and sources are credited.

## Data resources

### Data package title

Spatial and temporal distribution dataset of benthic macroalgae during the 2015-2016 tropical monsoonal cycle in Malaysia

### Resource link

https://cloud.gbif.org/asia/resource?r=dataset_macroalgae_johor&v=1.5

### Alternative identifiers

https://cloud.gbif.org/asia/resource?r=dataset_macroalgae_johor

### Number of data sets

1

### Data set 1.

#### Data set name

Spatial and temporal distribution dataset of benthic macroalgae during the 2015-2016 tropical monsoonal cycle in Malaysia

#### Data format

Darwin Core Archive (DwC-A)

#### Data format version

1.5

#### Description

This data paper presents the dataset on the inventory of macroalgae during the monsoonal storm cycle of 2015-2016 at the selected sites along the eastern coast of Johor, Malaysia. In particular, we focused on recording the occurrence of every species at the selected sites over the 14-months study period. Besides, substratum- and habitat-specificity of the macroalgae species is presented, which allows insights into assessing macroalgal abundances. In conclusion, the data serve as part of a larger assessment effort and the dataset synthesises the results of macroalgal diversity work done in the eastern coastal waters of Johor (Malaysia).

**Data set 1.** 

Column label	Column description
id	Same as OccurrenceID.
type	The nature or genre of the resource.
language	A language of the resource.
datasetName	The name identifying the dataset from which the record was derived.
basisofRecord	The specific nature of the data record.
occurenceID	An identifier for the Occurrence (as opposed to a particular digital record of the occurrence).
recordedBy	A list of names of peoples responsible for recording the original Occurrence.
individualCount	The number of individuals present at the time of the Occurrence.
organismQuantity	A number or enumeration value for the quantity of organisms.
organismQuantityType	The type of quantification system used for the quantity of organisms.
behaviour	The behaviour shown by the subject at the time the Occurrence was recorded.
occurenceStatus	A statement about the presence or absence of a Taxon at a Location.
preparations	A list (concatenated and separated) of preparations and preservation methods for a specimen.
disposition	The current state of a specimen with respect to the collection identified in collectionCode or collectionID.
occurenceRemarks	Comments or notes about the Occurrence.
eventDate	The date-time or interval during which an Event occurred.
habitat	A category or description of the habitat in which the Event occurred.
sampling protocol	The names of, references to, or descriptions of the methods or protocols used during an Event.
sampleSizeValue	A numeric value for a measurement of the size (time duration, length, area or volume) of a sample in a sampling event.
sampleSizeUnit	The unit of measurement of the size (time duration, length, area or volume) of a sample in a sampling event.
samplingEffort	The amount of effort expended during an Event.
eventRemarks	Comments or notes about the Event.
waterBody	The name of the water body in which the Location occurs.
country	The name of the country or major administrative unit in which the Location occurs.
stateProvince	The name of the next smaller administrative region than country (state, province, canton, department, region etc.) in which the Location occurs.
locality	The specific description of the place.
locationRemarks	Comments or notes about the Location.
decimalLatitude	The geographic latitude (in decimal degrees, using the spatial reference system given in geodeticDatum) of the geographic centre of a Location.
decimalLongitude	The geographic longitude (in decimal degrees, using the spatial reference system given in geodeticDatum) of the geographic centre of a Location.
geodeticDatum	The ellipsoid, geodetic datum or spatial reference system (SRS) upon which the geographic coordinates given in decimalLatitude and decimalLongitude are based.
coordinateUncertainty in metres	The horizontal distance (in metres) from the given decimalLatitude and decimalLongitude describing the smallest circle containing the whole of the Location.
identifiedBy	A list (concatenated and separated) of names of people, groups or organisations who assigned the Taxon to the subject.
dateIdentified	The date on which the subject was determined as representing the Taxon.
identificationReferences	A list of references (publication, global unique identifier, URI) used in the Identification.
identificationRemarks	Comments or notes about the Identification.
acceptedNameUsageID	An identifier for the name usage (documented meaning of the name according to a source) of the currently valid (zoological) or accepted (botanical) taxon.
scientificName	The full scientific name, with authorship and date information, if known.
parentNameUsage	The full name, with authorship and date information, if known, of the direct, most proximate higher-rank parent taxon (in a classification) of the most specific element of the scientificName.
kingdom	The full scientific name of the kingdom in which the taxon is classified.
phylum	The full scientific name of the phylum or division in which the taxon is classified.
class	The full scientific name of the class in which the taxon is classified.
order	The full scientific name of the order in which the taxon is classified.
family	The full scientific name of the family in which the taxon is classified.
taxonRank	The taxonomic rank of the most specific name in the scientificName.
scientificNameAuthorship	The authorship information for the scientificName formatted according to the conventions of the applicable nomenclaturalCode.
taxonomicStatus	The status of the use of the scientificName as a label for a taxon.
lifeStage	The age class or life stage of the Organisms at the time the Occurrence was recorded.
reproductiveCondition	The reproductive condition of the biological individuals is represented in Occurrence.

## Additional information

A total of 41 taxa were identified: three Groups (Rhodophyceae, Phaeophyceae and Clorophyceae), 17 Family (Rhodomelaceae, Lithophyllaceae, Corallinaceae, Pterocladiaceae, Gigartinaceae, Galaxauraceae, Gracilariaceae, Cystocloniaceae, Lomentariaceae, Dictyotaceae, Sargassaceae, Polyphysaceae, Caulerpaceae, Cladophoraceae, Boodleaceae, Ulvaceae and Valoniaceae) (Zainee and Rozaimi 2020). A description of number of taxa of each Order is presented in Suppl. material 7. Overall, our study sites in Tanjung Lompat had a higher number of species (31 species) per sites, followed by Telok Gorek (nine species) and Pantai Pasir Lanun (eight species). Pulau Mawar had the lowest number of species, five species (Zainee and Rozaimi 2020). Our findings presented significant changes in species composition due to the effects of the monsoon event.

## Supplementary Material

CEDD4AB0-0261-51BE-ABB3-52287412F1D1 10.3897/BDJ.10.e85676.suppl1 Supplementary material 1 Percent cover and frequency of macroalgae along the eastern coast of Johor Data type abundance Brief description Percent cover and frequency of macroalgae along the eastern coast of Johor were recorded from January 2015 to February 2016. The sub-ranges from 1-25 m refer to the points along the transect line (%C: percentage of cover; %F : percentage of frequency; NF: species not found along the replicate lines). This Table presents the spatial and temporal abundance of macroalgae from four different localities in the east coast of Johor, Malaysia. Percentage of cover and frequency of every species were recorded along the 25 m line transect which is grouped into 5 m intervals. File: oo_675307.csv https://binary.pensoft.net/file/675307 Zainee, N.F.A. and Rozaimi, M.

2F1F4FEA-1A23-5C9B-925B-1F6540D9665A 10.3897/BDJ.10.e85676.suppl2 Supplementary material 2 Raw data of cover and frequency of macroalgae recorded at Pantai Pasir Lanun Data type raw data - abundance Brief description Raw data of cover and frequency of macroalgae recorded at Pantai Pasir Lanun from January 2015 to February 2016 (TL1-TL9: transect numbers 1 to 9; NF: species not found along the replicate lines). This Table presents raw data of recorded species using five categories of multiplier, along the 25 m line transect and nine replications of transect line. File: oo_675308.csv https://binary.pensoft.net/file/675308 Zainee, N.F.A. and Rozaimi, M.

AAE3E9DE-5070-5072-8CD9-71C679EA8CD0 10.3897/BDJ.10.e85676.suppl3 Supplementary material 3 Raw data of cover and frequency of macroalgae recorded at Pulau Mawar Data type raw data - abundance Brief description Raw data of cover and frequency of macroalgae recorded at Pulau Mawar from January 2015 to February 2016 (TL1-TL9: transect number 1 to 9; NF: species not found along the replicate lines). This Table presents raw data of every species using five categories of multiplier, along the 25 m line transect and nine replications of transect line. File: oo_675309.csv https://binary.pensoft.net/file/675309 Zainee, N.F.A. and Rozaimi, M.

2EC08EE9-D814-5808-9180-26E56035698E 10.3897/BDJ.10.e85676.suppl4 Supplementary material 4 Raw data of cover and frequency of macroalgae recorded at Telok Gorek Data type raw data - abundance Brief description Raw data of cover and frequency of macroalgae recorded at Telok Gorek from January 2015 to February 2016 (TL1-TL9: transect number 1 to 9; NF: species not found along the replicate lines). This Table presents raw data of every species using five categories of multiplier, along the 25 m line transect and nine replications of transect line. File: oo_675310.csv https://binary.pensoft.net/file/675310 Zainee, N.F.A. and Rozaimi, M.

AEA66FC1-2B85-5347-A0D1-DAC929D04B6C 10.3897/BDJ.10.e85676.suppl5 Supplementary material 5 Raw data of cover and frequency of macroalgae recorded at Tanjung Lompat Data type raw data - abundance Brief description Raw data of cover and frequency of macroalgae recorded at Tanjung Lompat from January 2015 to February 2016 (TL1-TL9: transect number 1 to 9; NF: species not found along the replicate lines). This Table presents raw data of every species using five categories of multiplier, along the 25 m line transect and nine replications of transect line. File: oo_675311.csv https://binary.pensoft.net/file/675311 Zainee, N.F.A. and Rozaimi, M.

0DAF1B89-73F5-5A06-BF20-4B1CC2968321 10.3897/BDJ.10.e85676.suppl6 Supplementary material 6 The 5 categories of multiplier used by Saito and Atobe (1970) Data type calculation for percent cover and frequency Brief description The 5 categories of multiplier used by Saito and Atobe (1970) to represent surface area covered by macroalgal species on a small sub-quadrat. This Table illustrates the calculation of the percentage of cover and frequency of macroalgae. File: oo_669566.pdf https://binary.pensoft.net/file/669566 Zainee, N.F.A. and Rozaimi, M.

73182187-D347-5D16-AEAD-D026064495DD 10.3897/BDJ.10.e85676.suppl7 Supplementary material 7 Taxonomic literature of marine macroalgae found in the eastern coast of Johor, Malaysia Data type taxonomic literature Brief description This Table presents the taxonomy hierarchy of every macroalgae species found in the east coast of Johor, Malaysia. File: oo_675312.csv https://binary.pensoft.net/file/675312 Zainee, N.F.A. and Rozaimi, M.

## Figures and Tables

**Table 1.** Coordinate, types of shore, coastline feature, types of vegetation and date of sampling of every study site.

Descriptions	Tanjung Lompat	Telok Gorek	Pulau Mawar	Pantai Pasir Lanun
Latitude, Longitude	1°36'10"N, 104°15'17"E	2°18' 37"N, 103°57'31"E	2°37'08"N, 103°47'01"E	2°38'52"N, 103°45'29"E
Types of shore	sandy, rocky	sandy	sandy	sandy, rocky
Coastline feature	bay	foreland	foreland	foreland
Vegetation	-	mangrove	mangrove	-
Date of sampling	10-Jan-15	21-Jan-15	22-Jan-15	23-Jan-15
	8-Feb-15	19-Feb-15	20-Feb-15	21-Feb-15
	10-Mar-15	20-Mar-15	21-Mar-15	22-Mar-15
	8-Apr-15	19-Apr-15	20-Apr-15	21-Apr-15
	8-May-15	19-May-15	20-May-15	21-May-15
	8-Jun-15	17-Jun-15	18-Jun-15	19-Jun-15
	6-Jul-15	31-Jul-15	31-Jul-15	31-Jul-15
	29-Aug-15	30-Aug-15	30-Aug-15	31-Aug-15
	1-Aug-15	14-Sep-15	14-Sep-15	15-Sep-15
	26-Oct-15	27-Oct-15	28-Oct-15	29-Oct-15
	14-Nov-15	25-Nov-15	26-Nov-15	27-Nov-15
	15-Dec-15	26-Dec-15	27-Dec-15	28-Dec-15
	14-Jan-16	26-Jan-16	27-Jan-16	28-Jan-16
	13-Feb-16	24-Feb-16	25-Feb-16	26-Feb-16

**Table 2.** Life form of macroalgae and mode of substrate attachment (1 indicates the life-form of each species; N = natural substratum; A = artificial substratum; a = epilithic; b = epipsamonic; c = epizoic; d = epipelic; e = epiphytic; f = ropes; g = gunny fibres; h = fishing net).

Species list	N_a	N_b	N_c	N_d	N_e	A_f	A_g	A_h
**Green algae**								
*Caulerparacemosa*	1	1						
*Cladophoropsismembranacea*	1	1		1	1			
*Chaetomorphaaerea*	1	1	1	1	1	1	1	
*C.crassa*	1							
*C.ligustica*	1	1	1	1	1	1	1	
*C.linum*	1	1	1	1	1	1	1	
*C.minima*	1	1	1	1	1	1	1	
*Cladophorastimpsonii*	1							
*C.vagabunda*	1							
*Valoniaaegagropila*	1							
*Acetabulariaacetabulum*	1							
*Ulvaclathrata*	1		1	1				
*U.intestinalis*	1	1						
**Brown algae**								
*Dictyopterisdelicatula*	1							
*Canistrocarpuscervicornis*	1				1			
*Dictyotamertensii*	1							
*D.dichotoma*	1							
*Padina australis*	1	1						
*P.boergesenii*								
*P.minor*	1	1						
*Sargassumoligocystum*	1							1
*S.paniculatum*	1							1
*S.polycystum*	1							
*S.microcystum*	1							
*S.tenerrimum*		1						
**Red algae**								
*Acanthophoramuscoides*		1						
*A.spicifera*	1							
*Polysiphoniacoacta*		1						
*Amphiroafragilissima*	1	1						
*Janiaadhaerens*	1							
*Pterocladiellacaloglossoides*	1	1						
*Chondruscrispus*	1							
*Hypneacervicornis*		1			1			
*H.spinella*		1						
*Gracilariaarcuata*	1	1				1		
*G.blodgetti*	1	1						
*G.bursa-pastoris*	1	1						
*Crassiphycuschangii*		1						
*G.coronopifolia*	1	1				1		
*G.salicornia*	1	1						
*Galaxaurarugosa*	1							
*Ceratodictyonintricatum*	1	1			1			
