# Supplementary material for: Spatial and temporal distribution dataset of benthic macroalgae during the 2015-2016 tropical monsoonal cycle in Malaysia
Source: Biodivers Data J. 2022 Jul 26;10:e85676. doi: 10.3897/BDJ.10.e85676 (PMC9848546; doi:10.3897/BDJ.10.e85676)
Supplement: Supplementary material 6 — The 5 categories of multiplier used by Saito and Atobe (1970) [file bdj-10-e85676-s006.pdf]

Supplementary Table S5. The 5 categories of multiplier used by Saito and Atobe (1970) to represent surface area covered by macroalgal species on a small sub-quadrat.

Algal cover	Illustration	Multiplier (C_n)
Covering ½ to 1 of substratum surface	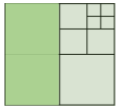	3.0
Covering ¼ to ½ of substratum surface	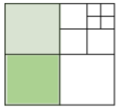	1.5
Covering $^1/_8$ to ¼ of substratum surface	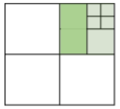	0.75
Covering $^1/_{16}$ to $^1/_8$ of substratum surface	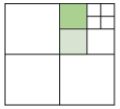	0.375
Covering less than $^1/_{16}$ of substratum surface	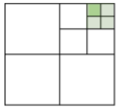	0.1875
